# Neurogenesis Response of Middle-Aged Hippocampus to Acute Seizure Activity

**DOI:** 10.1371/journal.pone.0043286

**Published:** 2012-08-17

**Authors:** Ashok K. Shetty, Bharathi Hattiangady, Muddanna S. Rao, Bing Shuai

**Affiliations:** 1 Research Service, Veterans Affairs Medical Centers of Durham, North Carolina, and Temple, Texas, United States of America; 2 Institute for Regenerative Medicine, Texas A&M Health Science Center College of Medicine at Scott & White, Temple, Texas, United States of America; 3 Department of Molecular and Cellular Medicine, Texas A&M Health Science Center College of Medicine, College Station, Texas, United States of America; 4 Department of Surgery (Neurosurgery), Duke University Medical Center, Durham, North Carolina, United States of America; Beijing Institute of Radiation Medicine, China

## Abstract

Acute Seizure (AS) activity in young adult age conspicuously modifies hippocampal neurogenesis. This is epitomized by both increased addition of new neurons to the granule cell layer (GCL) by neural stem/progenitor cells (NSCs) in the dentate subgranular zone (SGZ), and greatly enhanced numbers of newly born neurons located abnormally in the dentate hilus (DH). Interestingly, AS activity in old age does not induce such changes in hippocampal neurogenesis. However, the effect of AS activity on neurogenesis in the middle-aged hippocampus is yet to be elucidated. We examined hippocampal neurogenesis in middle-aged F344 rats after a continuous AS activity for >4 hrs, induced through graded intraperitoneal injections of the kainic acid. We labeled newly born cells via daily intraperitoneal injections of the 5′-bromodeoxyuridine (BrdU) for 12 days, commencing from the day of induction of AS activity. AS activity enhanced the addition of newly born BrdU+ cells by 5.6 fold and newly born neurons (expressing both BrdU and doublecortin [DCX]) by 2.2 fold to the SGZ-GCL. Measurement of the total number of DCX+ newly born neurons also revealed a similar trend. Furthermore, AS activity increased DCX+ newly born neurons located ectopically in the DH (2.7 fold increase and 17% of total newly born neurons). This rate of ectopic migration is however considerably less than what was observed earlier for the young adult hippocampus after similar AS activity. Thus, the plasticity of hippocampal neurogenesis to AS activity in middle age is closer to its response observed in the young adult age. However, the extent of abnormal migration of newly born neurons into the DH is less than that of the young adult hippocampus after similar AS activity. These results also point out a highly divergent response of neurogenesis to AS activity between middle age and old age.

## Introduction

Production of new neurons through proliferation of neural stem/progenitor cells (NSCs) and neuronal differentiation of newly born cells occurs all through life in the hippocampus of nearly all mammals [1]–[4]. A large body of research in animal models suggests that a significant fraction of newly born neurons (i.e. dentate granule cells) integrate into the hippocampal circuitry and participate in functions such as learning, memory and mood [5]–[12]. However, the extent of hippocampal neurogenesis varies conspicuously in response to alterations in the dentate gyrus (DG) microenvironment. For example, increased levels of NSC mitogenic factors (such as fibroblast growth factor-2 [FGF-2], brain-derived neurotrophic factor [BDNF], insulin-like growth factor-1 [IGF-1] and vascular endothelial growth factor [VEGF]), enhanced physical activity, caloric restriction, environmental enrichment, and increased neural activity considerably enhance the extent of hippocampal neurogenesis [13]–[25]. In contrast, aging is a negative regulator of hippocampal neurogenesis, which is typified by an increased quiescence of NSCs likely due to age-related adverse changes in their microenvironment ([26]–[30]; however, see [31]). Thus, the DG microenvironment appears to be one of the major factors that regulate the extent of hippocampal neurogenesis.

The young hippocampus typically responds to insults such as stroke and acute seizure (AS) activity with considerable enhancements in NSC mitogenic factors and increased hippocampal neurogenesis, which likely reflect a compensatory plasticity in response to injury [32]–[39]. However, it is unclear whether such injury-induced increases in hippocampal neurogenesis are beneficial or detrimental to the hippocampal function. While studies in certain brain injury models suggest that such plasticity is valuable for diminishing impairments in cognitive function after brain injury [40]–[42], studies in seizure models suggest that such plasticity (particularly the abnormal migration and synaptic connectivity of newly born neurons) contributes to epileptogenesis and the development of chronic epilepsy [43; however, see 44, 45]. On the other hand, the aged hippocampus responds to insults such as stroke with either mild or no increase in neurogenesis [34]. Furthermore, the aged hippocampus fails to increase the extent of neurogenesis in response to AS activity because of predominantly glial differentiation of newly born cells in the aged DG after AS activity [46]. Moreover, the loss of neurogenic response in the aged hippocampus to AS activity is typically associated with severe memory impairments and chronic epilepsy [47]. Thus, the neurogenic response of the hippocampus diverges considerably between the young adult age and the old age. Additionally, the loss of neurogenic response may be contributing to the severe cognitive dysfunction and chronic epilepsy observed in the aged population after AS activity.

The neurogenic response of the middle-aged hippocampus to AS activity is currently unknown. This is an important issue because the age-related increased quiescence of NSCs, decreases in the basal concentration of NSC proliferation factors, and diminished neurotrophic response after injury, become apparent as early as middle age in the hippocampus [29], [30], [48], [49]. Considering the above, it is of interest to ascertain whether the response of hippocampal neurogenesis to AS activity in middle age is akin to its response observed in the young adult age or to what was seen in the old age. We investigated the extent and pattern of hippocampal neurogenesis after a continuous AS activity (stages III–V seizures) for over 3 hrs in the middle-aged (12-months old) F344 rats. The seizures were induced through 2–4 graded intraperitoneal injections of the kainic acid (KA). Newly born cells in the DG were labeled via daily intraperitoneal injections of the 5′-bromodeoxyuridine (BrdU) for 12 days, commencing from the day of AS activity. Newly born cells and neurons in the subgranular zone (SGZ) and the granule cell layer (GCL) were quantified at 24 hours after the last BrdU injection (equivalent to 13 days post-injury) using BrdU and doublecortin (DCX) immunostaining, BrdU-DCX dual immunofluorescence and confocal microscopy, and the optical fractionator cell counting method [50], [51]. Furthermore, to ascertain the extent of aberrant migration of newly born neurons, numbers of ectopically placed newly born DCX-positive (DCX+) neurons were measured in the dentate hilus (DH).

## Results

### AS Activity and Hippocampal Neurodegeneration with KA Injections

**Figure 1:**
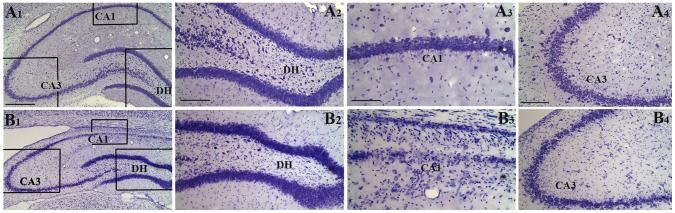
Comparison of the hippocampal cytoarchitecture via Nissl staining between an intact middle-aged animal (A1) and a middle-aged animal that underwent acute seizure (AS) activity (B1). The hippocampal section illustrated in B1 is visualized at 13 days after kainic acid induced AS activity. Figures A2–A4 and B2–B4 show magnified views of marked regions of the DH (A2, B2), the CA1 subfield (A3, B3) and the CA3 subfield (A4, B4) from figures A1 and B1 respectively. Note that considerable neurodegeneration is apparent in the DH (B2), the CA3c subregion (B2) and the CA1 cell layer (B3) of the middle-aged animal that underwent AS activity. Scale bar, A1, B1 = 500 µm; A2, A4, B2, B4 = 200 µm; A3, B3 = 100 µm.

Acute seizure activity was induced in middle-aged (12-months old) F344 rats through 2–4 graded intraperitoneal injections of the excitotoxin KA (3 mg/Kg body weight [b.w.]/hour). Seizures were observed for over 4 hours after 2–4 injections of KA. The behavioral seizures that manifested as unilateral forelimb clonus (stage III seizures), bilateral forelimb clonus (stage IV seizures), or bilateral forelimb clonus with rearing and falling (stage V seizures) were quantified in seven animals. The average numbers of seizures varied from 7 seizures (Mean ± SEM, 7.0±0.3) in the first hour, ∼12 seizures (11.9±0.6) in the second hour, ∼19 seizures in the third hour (18.8±0.92) and ∼20 seizures (19.6±1.5) in the fourth hour after the onset of seizure activity. The average duration of individual seizures was ∼26 seconds (26.3±1.0) in the first hour, ∼44 seconds (43.5±1.1) in the second hour, ∼49 seconds (49±1.3) in the third hour, and ∼53 seconds (53.1±2.4) in the fourth hour after the onset of seizure activity. Kainic acid induced AS activity caused significant neurodegeneration in the hippocampus and extrahippocampal regions when examined 13 days after AS activity. In the hippocampus, bilateral neuronal cell loss was evident in the DH, CA1 pyramidal cell layer and CA3c sub region of the CA3 pyramidal cell layer (Fig. 1 [B1–B4]), in comparison to the intact age-matched hippocampus (Fig. 1 [A1–A4]). However, the dentate granule cell layer did not display any apparent loss of neurons (Fig. 1 [B1, B2]). Thus, the extent of hippocampal neurodegeneration after KA-induced AS activity in middle-aged animals appeared akin to what was reported earlier for young adult and aged animals [46], [52].

### Addition of Newly Born Cells to the SGZ-GCL after AS Activity

**Figure 2:**
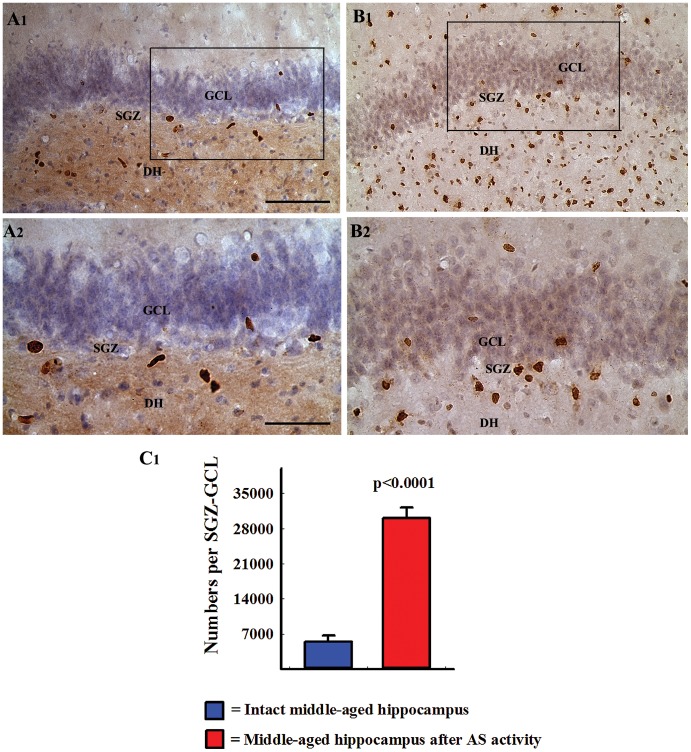
Comparison of the distribution of newly generated cells in the dentate gyrus (DG) at 24 hours after 12 daily injections of 5′-bromodeoxyuridine (BrdU) between an intact middle-aged animal (A1) and a middle-aged animal that underwent acute seizure (AS) activity (B1). The cells are visualized through BrdU immunostaining and hematoxylin counterstaining. A2 and B2 are magnified views of marked regions from A1 and B1. Note that the density of newly born cells in the subgranular zone-granule cell layer (SGZ-GCL) increases in the middle-aged rat after AS activity (B1, B2), in comparison to the age-matched naïve rat (A1 and A2). Also note that the dentate hilus (DH) shows increased density of BrdU immunopositive cells in the rat that underwent AS activity (B1). Scale bar, A1 and B1 = 100 µm; A2 and B2 = 50 µm. The bar chart in C1 compares absolute numbers of newly born cells added over a period of 12 days to the SGZ-GCL between intact middle-aged rats and middle-aged rats that underwent AS activity (n = 5/group). Note that, AS activity considerably increases the numbers of newly born cells in the SGZ-GCL of middle-aged rats.

Addition of newly born cells to the SGZ-GCL over a period of 12 days after AS activity was examined via BrdU immunostaining at 24 hours after the last of twelve daily BrdU injections (n = 5/group). This demonstrated newly generated cells in the SGZ-GCL throughout their antero-posterior extent (Fig. 2 [A1–B2]). In comparison to the SGZ-GCL of age-matched intact control animals, SGZ-GCL of animals that underwent AS activity exhibited a clear increase in the density of newly born BrdU-positive (BrdU+) cells (Fig. 2 [A1–B2]). The degenerated regions (the DH and the CA3 pyramidal cell layer) also showed a large number of BrdU+ cells (Fig. 2 [B1]). Quantification of the numbers of BrdU+ cells revealed enhanced addition of newly born cells to the SGZ-GCL after AS activity (5.6 folds increase, p<0.001, Fig. 2 [C1]).

### Extent of Differentiation of Newly Born Cells in the SGZ-GCL into Immature DCX+ Neurons after AS Activity

**Figure 3:**
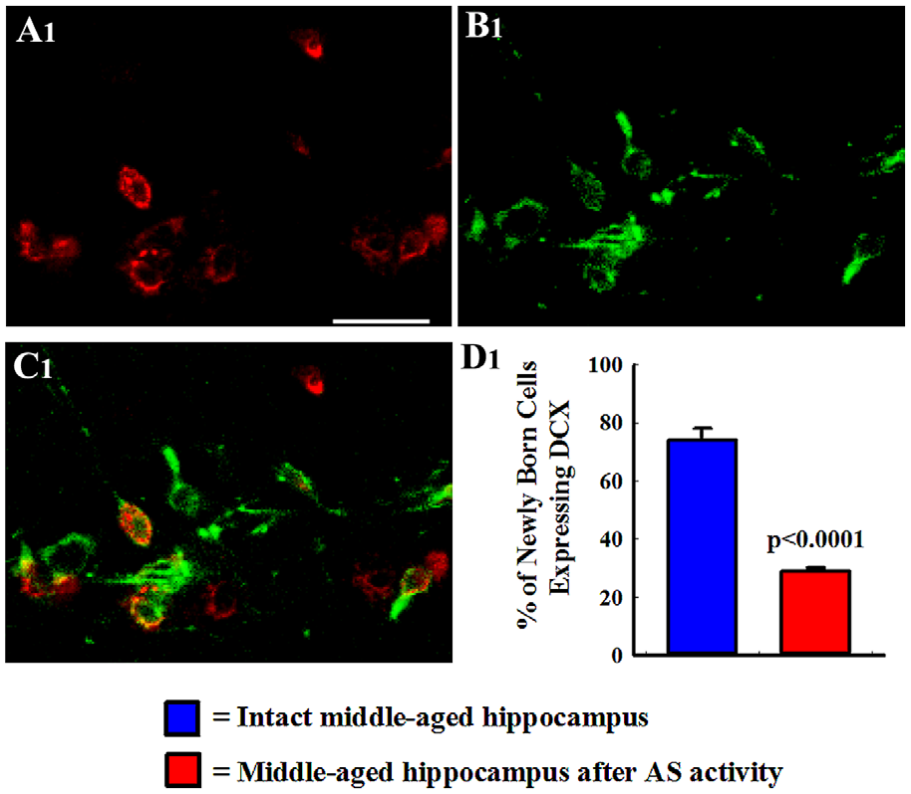
Neuronal differentiation of newly born cells in the subgranular zone-granule cell layer (SGZ-GCL). Analyses were performed at 24 hours after the last of twelve daily injections of 5′-bromodeoxyuridine (BrdU) and visualized through BrdU and doublecortin (DCX) dual immunofluorescence and confocal microscopy. Figures A1–C1 show examples of newly born (BrdU+) cells (red nuclei) that are positive for DCX (green fluorescence in the cytoplasm of the soma and dendrites). The bar chart in D1compares percentages of BrdU+ cells that express DCX (n = 5/group). Note that AS activity diminishes the overall neuronal differentiation of newly born cells. Scale bar, A1–B3 = 20 µm.

Dual immunofluorescence for BrdU and DCX and confocal microscopic analyses revealed neurons among newly born BrdU+ cells in the SGZ-GCL (Fig. 3 [A1–C1]). The BrdU expression was found in the nucleus whereas DCX staining was observed in the soma and dendrites of newly born neurons (Fig. 3 [A1–C1]). Doublecortin, an excellent marker of immature neurons in the DG, is typically expressed within 3 hours after birth in neuronally committed newly born cells [53], and serves as a marker for at least two weeks after birth [50]. Quantification of BrdU+ cells expressing DCX revealed that AS activity in middle-aged animals reduced the fraction of newly born cells that differentiate into neurons (n = 5/group). The overall neuronal differentiation of newly born cells was reduced to 29.0±1.5% after AS activity from 74±1.9% observed in intact animals (p<0.0001, Fig. 3 [D1]).

### Extent of Initial Neurogenesis after AS Activity

**Figure 4:**
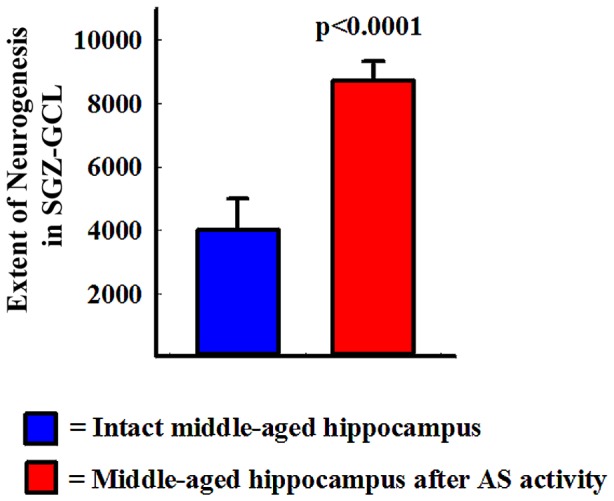
Comparison of absolute numbers of newly born neurons added over a period of 12 days to the subgranular zone-granule cell layer (SGZ-GCL) between intact middle-aged rats and middle-aged rats that underwent acute seizure (AS) activity. The extent of neurogenesis was calculated by using absolute numbers of newly born cells and percentages of newly born cells that differentiate into neurons in both groups. Note that, AS activity considerably increases the extent of neurogenesis in the SGZ-GCL of middle-aged rats (n = 5/group).

Using data such as the total number of BrdU+ newly born cells and the percentage of newly born cells that differentiate into DCX+ neurons, we measured the extent of neurogenesis in the SGZ-GCL after AS activity (n = 5/group). The extent of hippocampal neurogenesis was clearly increased after AS activity (2.2 folds increase, p<0.0001, Fig. 4). Thus, despite a reduced conversion of newly born cells into neurons, AS activity enhanced the extent of neurogenesis in the SGZ-GCL of middle-aged animals through a much greater increase in the production of new cells.

### Numbers of DCX+ New Neurons after AS Activity

**Figure 5:**
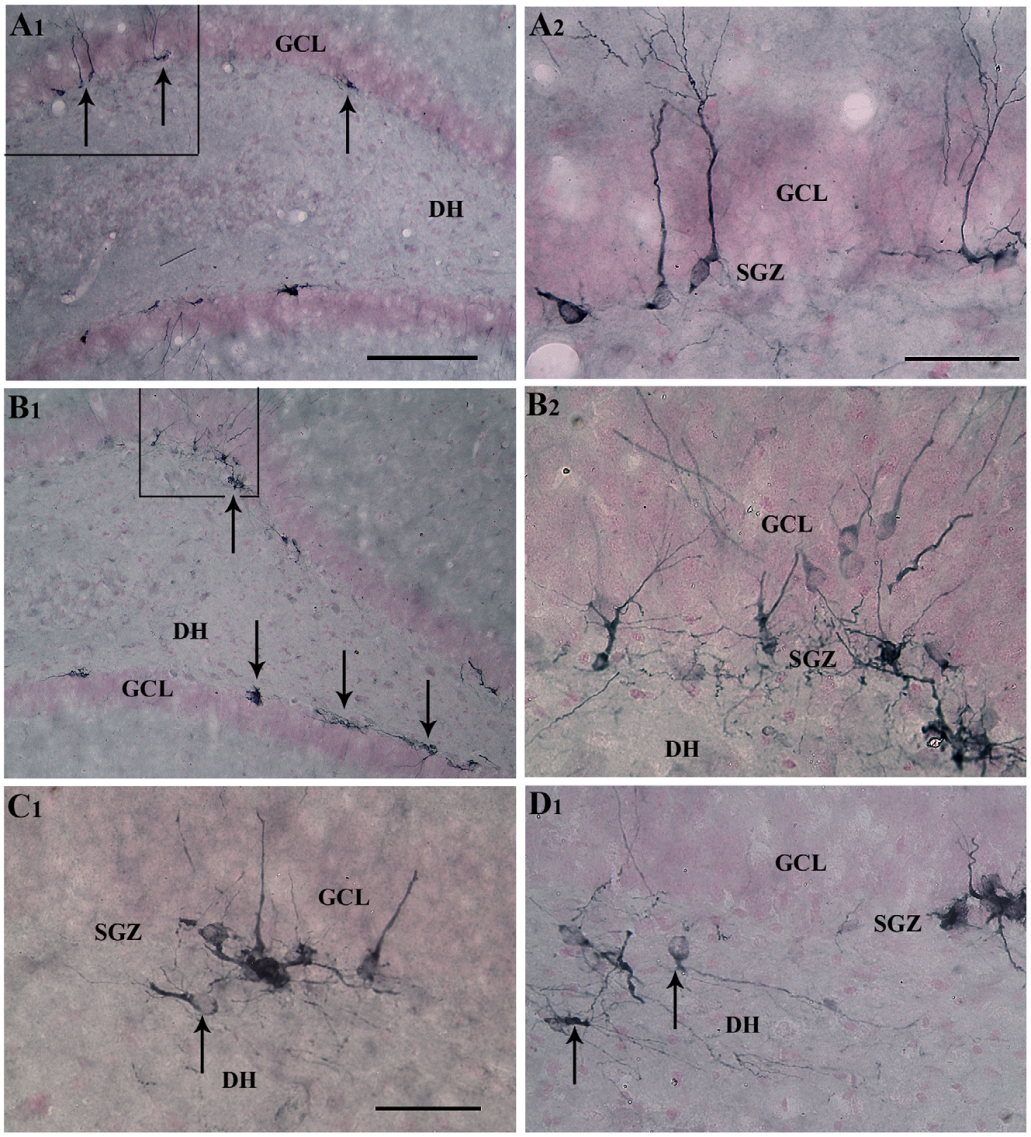
Comparison of the distribution of newly born neurons in the subgranular zone-granule cell layer (SGZ-GCL) between an intact middle-aged animal (A1) and a middle-aged animal that underwent acute seizure (AS) activity (B1). The sections were visualized through doublecortin (DCX) immunostaining. A2 and B2 are magnified views of marked regions from A1 and B1. Arrows in A1 and B1 denote clusters of newly born neurons. Note that the overall density of newly born neurons increases in the middle-aged rat after AS activity (B1, B2), in comparison to the age-matched naïve rat (A1, A2). Figures C1 and D1 show ectopically placed newly born neurons in the dentate hilus (DH; arrows) of an intact middle-aged rat (C1) and a middle-aged rat that underwent AS activity (D1). Scale bars, A1, B1 = 200 µm; A2, B2, C1, D1 = 50 µm.

**Figure 6:**
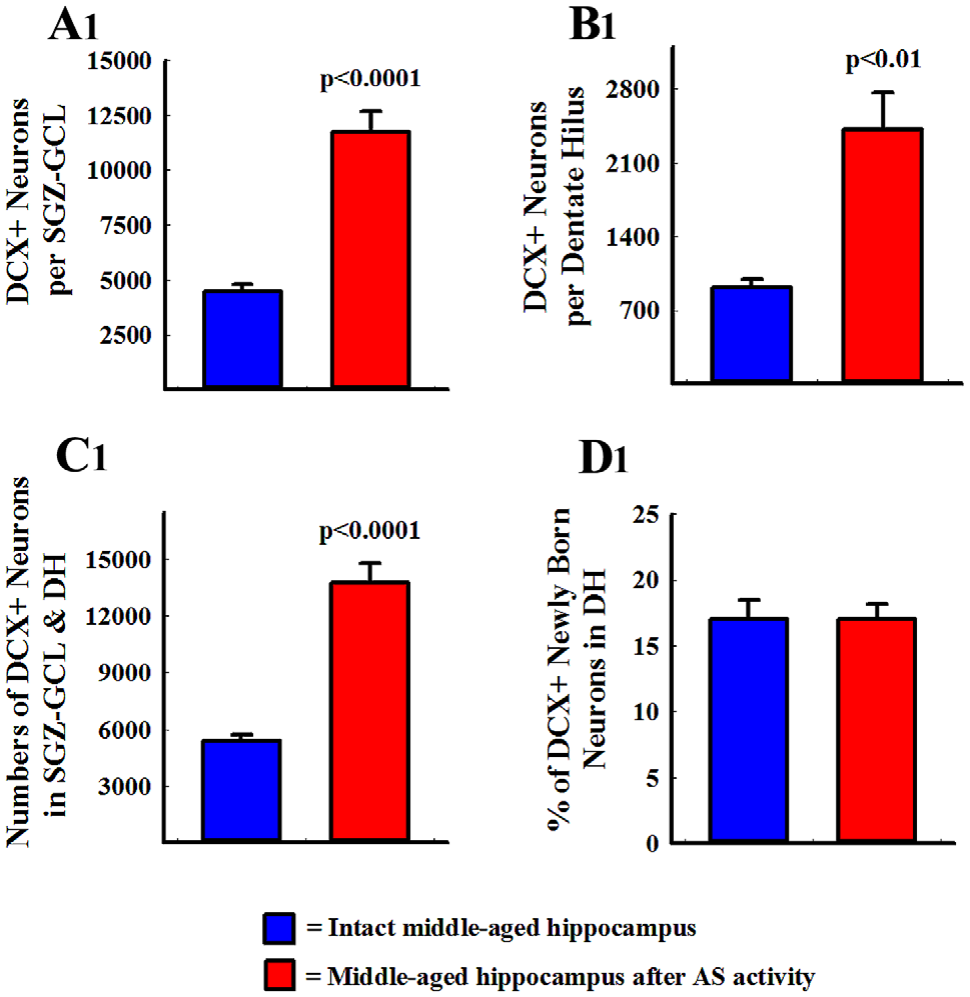
Comparison of the numbers of doublecortin-positive (DCX+) newly born neurons between intact middle-aged rats (n = 5) and middle-aged rats (n = 5) that underwent acute seizure (AS) activity in the subgranular zone-granule cell layer (SGZ-GCL; A1), the dentate hilus (DH; B1), the SGZ-GCL + DH (C1). The numbers of newly born neurons are proportionately increased after AS activity in the SGZ-GCL (A1) and the DH (B1). The bar chart in D1 compares percentages of DCX+ neurons that are located in the DH between the two groups. Note that, the percentage of DCX+ neurons that migrate into the DH are comparable between intact middle-aged rats and middle-aged rats that underwent AS activity.

We examined DCX+ newly born neurons in the SGZ-GCL and the DH as an additional measure of the status of DG neurogenesis after AS activity (Fig. 5 [A1–D1]; n = 5/group). An increased density of DCX+ newly born neurons was observed after AS activity in the SGZ-GCL (Fig. 5 [B1, B2]), in comparison to their counterparts in age-matched intact control animals (Fig. 5 [A1, A2). The DH showed DCX+ newly born neurons in both intact and post-seizure conditions (Fig. 5 [C1, D1]). However, the density of such neurons was greater after AS activity, reflecting an increased abnormal migration of newly born neurons with AS activity (Fig. 5 [D1]). Most DCX+ newly born neurons that migrated into the DH exhibited horizontally oriented dendrites or dendrites that projected mostly towards the DH (i.e. basal dendrites; Fig. 5 [C1, D1]). In addition, some newly born neurons located in the SGZ-GCL exhibited long basal dendrites (Fig. 5 [C1, D1]), as reported previously for young adult animals after AS activity [54], [55]. We separately measured the numbers of DCX+ neurons in the SGZ-GCL and the DH (Fig. 6 [A, B]). This demonstrated that AS activity increased the numbers of newly born DCX+ neurons in the SGZ-GCL by 2.6 folds (p<0.0001, Fig. 6 [A]). This level of enhancement is akin to the 2.2 folds increase observed in the extent of neurogenesis described above. This is not surprising because our previous study has shown that a vast majority of DCX+ neurons represent neurons that were born during the 12 days prior to euthanasia [50]. The DH exhibited 2.7 folds increase in the number of DCX+ neurons (p<0.01, Fig. 6 [B]). When numbers of newly born neurons in the SGZ-GCL and the DH were combined, the DG displayed 2.6 folds increase in numbers of newly born neurons after AS activity (p<0.0001; Fig. 6 [C]). However, percentages of total numbers of DCX+ neurons that migrate into the DH were comparable (17%) between intact rats and rats that underwent AS activity because AS activity proportionately increased the numbers of newly born neurons in both SGZ-GCL and the DH. Thus, the overall neurogenic response of the middle-aged hippocampus to AS activity appeared closer to the response of the young adult hippocampus than the response of the aged hippocampus to AS activity reported earlier [35], [37], [46].

## Discussion

This study provides novel evidence that NSCs in the SGZ of the middle-aged hippocampus retain their ability to enhance neurogenesis in response to AS activity. Our earlier study, by comparing the response of NSCs to AS activity in the young adult and aged hippocampi, has suggested that the ability of NSCs to enhance neurogenesis in response to AS activity is lost with aging [46]. However, the current results demonstrate that major modifications in neurogenesis response to AS activity do not occur at least until the middle age. Interestingly, the overall increase observed in normal neurogenesis in response to AS activity in the middle-aged hippocampus matched the extent of increase seen earlier in the young adult hippocampus after similar AS activity [46]. However, this reaction greatly differed from the lack of neurogenesis response observed in the aged hippocampus after AS activity [46].

### Effect of Aging on AS Activity Mediated Normal Hippocampal Neurogenesis

A normal pattern of hippocampal neurogenesis involves continuous addition of newly born neurons to the GCL through proliferation of hippocampal NSCs in the SGZ, and neuronal differentiation and migration of newly born cells into the GCL. This pattern of neurogenesis in the young or adult hippocampus is highly sensitive to AS activity, which has been authenticated by the observation of increased addition of newly born neurons to the GCL by hippocampal NSCs in several animal models of epilepsy [35]–[39], [55]. Our earlier study performed in young adult F344 rats demonstrated that AS activity enhances the addition of newly born cells to the SGZ-GCL by 2.3 folds but not in the aged (24-month old F344 rat) hippocampus [46]. However, it was unclear whether the loss of neurogenesis response to AS activity would occur as early as middle age or just restricted to the very old age. The current study addressed this issue and demonstrates that NSCs in the SGZ of the middle-aged hippocampus retain their ability to enhance normal neurogenesis in response to AS activity. These results highlight that major alterations in neurogenesis response to AS activity do not occur at least until the middle age. The extent of neuronal differentiation of newly born cells after AS activity in the middle-aged hippocampus (29%) was however midway between the extent seen in the young adult hippocampus (68%) and the aged hippocampus (9%) after similar AS activity [46]. Nevertheless, the rate of conversion of newly born cells into neurons was adequate for enhancing the overall extent of neurogenesis by 2.2 folds because the overall addition of new cells to the SGZ-GCL exhibited 5.6 folds increase after AS activity. The results also imply that age-related changes in the amount of neurogenesis in response to AS activity are not due to a lack of proliferation of NSCs with aging but rather due to changes in the neuronal differentiation of newly born cells, which is likely a consequence of adverse changes in the microenvironment after AS activity with aging. Interestingly, such adverse changes in microenvironment in response to AS activity appear to occur progressively during the course of aging.

The precise role of increased extent of normal neurogenesis after AS activity is yet to be elucidated. However, a previous study shows that newly born neurons added to the GCL after AS-activity have reduced excitability than newly born neurons that are added to the GCL under normal conditions [44]. This has resulted in a suggestion that newly added granule cells to the GCL after AS activity attempt to ease the AS-activity induced hyperexcitability in the DG [44]. From this perspective, increased level of normal pattern of neurogenesis after AS activity appears beneficial for reducing the DG hyperexcitability and spontaneous recurrent seizures observed in the chronic phase after AS activity. Furthermore, recruitment of greater numbers of newly born granule cells into the hippocampal circuitry may also reduce the overall cognitive dysfunction in the chronic phase after AS activity because newly born granule cells that integrate into the hippocampal circuitry are believed to strengthen the learning and memory function [7]–[12]. If these premises are accurate, one would then expect the aged animals to display greater numbers of spontaneous seizures and severe cognitive dysfunction than young adult animals after similar AS activity because AS activity has no effect on normal neurogenesis in the aged hippocampus but greatly increases normal neurogenesis in the young adult hippocampus [46]. Indeed, our recent study shows that similar levels of AS activity leads to an increased frequency and intensity of spontaneous recurrent seizures and greater cognitive dysfunction in aged rats than in young adult rats [47]. Thus, it is likely that increased levels of normal hippocampal neurogenesis after AS activity are beneficial for reducing both spontaneous recurrent seizures and cognitive dysfunction emerging in the chronic phase after AS activity. However, this compensatory reaction occurring from the plasticity of NSCs in response to seizures is not sufficient for blocking the evolution of AS activity induced initial hippocampal insult into chronic epilepsy in young adult animals [47]. This is likely due to the emergence of multiple other epileptogenic changes after the initial hippocampal insult [54], [56]–[63].

### Effect of Aging on AS Activity Mediated Abnormal Hippocampal Neurogenesis

Abnormal pattern of hippocampal neurogenesis is characteristically seen after AS activity or brain injury [35], [64], [65]. This is typified by aberrant migration of significant fractions of newly born neurons into the DH, which is predominantly conspicuous in the early phase after a brain insult. In this study, AS activity increased abnormal neurogenesis by 2.7 folds in the middle-aged hippocampus, which closely parallels 2.2 folds increase in normal neurogenesis described above. This implies a balanced increase in the numbers of newly born neurons in the GCL and the DH after AS activity in middle-aged animals. This pattern is different from what was seen in the young adult hippocampus but closer to the situation in the aged hippocampus, as AS activity increases abnormal neurogenesis by 10 folds in the young adult hippocampus and 1.7 folds in the aged hippocampus [46]. There is a strong suggestion in the current literature that aberrant integration of newly born granule cells in the DH contributes to the development of spontaneous recurrent seizures in the chronic phase of epilepsy [43], [65]–[71]. Based on the above, one would expect the aged and middle-aged animals to display lower numbers of spontaneous recurrent seizures than young adult animals after similar AS activity because AS activity minimally increases the actual numbers of abnormally displaced granule cells in the DH of the middle-aged and aged hippocampi but greatly increases the population of such neurons in the young adult hippocampus [46]. However, comparison of spontaneous recurrent seizures between aged and young adult animals after AS activity in a recent study reveals that the opposite is true [47]. Thus, a cause-effect relationship between abnormal neurogenesis and incidences of spontaneous seizures seems unlikely. A study in a kindling model of epilepsy also supports this conclusion [45]. Nevertheless, based on the aberrant integration of newly born neurons that migrate into the DH [65]–[71], some contribution of abnormal neurogenesis to spontaneous recurrent seizures cannot be ruled out particularly in the young adult hippocampus.

### Plasticity of NSCs in the Middle-aged Hippocampus to Different Types of Insults

Inability of NSCs in the aged hippocampus to up-regulate normal neurogenesis following various types of insults is well documented in most studies of aged animals and human samples ([34], [38], [46], [72], [73]; but see [74]). In contrast, the response of NSCs in the middle-aged hippocampus to various insults is mostly positive in terms of enhancing neurogenesis, be it a model of stroke [75], hippocampal deafferentation [51] or seizures (the current study). However, a few studies have also suggested inability of NSCs in the middle-aged hippocampus to up-regulate neurogenesis in response to excitotoxic cell death of CA3 pyramidal neurons [38] and chronic cerebral hypoperfusion [76]. This implies that NSC plasticity in the middle-aged hippocampus depends on the type of hippocampal injury. Considerable damage to the middle-aged hippocampus, particularly a significant loss of efferent target neurons of the newly generated neurons such as CA3 pyramidal neuron loss occurring with excitotoxic injury, is associated with loss of NSC plasticity [38]. On the other hand, insults involving a moderate or no loss of CA3 pyramidal neurons such as after AS activity, stroke, and deafferentation stimulate NSC plasticity and enhance neurogenesis in the middle-aged hippocampus [51, 75 and the current study]. Differential response of neurogenesis in the middle-aged hippocampus to brain insults may also be due to diverging response of neurotrophic factors that positively regulate neurogenesis such as BDNF, FGF-2 and VEGF to various types of hippocampal injury. This possibility is also supported by the earlier findings that CA3 region excitotoxic injury in the middle-aged hippocampus does not increase the concentration of BDNF to levels seen in the young adult hippocampus after similar injury [49].

## Materials and Methods

### Animals

Middle-aged (12-months old) animals obtained from the National Institutes of Aging colony of F344 rats (Harlan Sprague-Dawley, Indianapolis, IN) were used in this study. A week after arrival, animals were classified into two groups: a control group (n = 5) where animals were maintained without any intervention, and a seizure group where animals received graded intraperitoneal injections of KA (n = 8). All experiments were performed as per the animal protocol approved by the animal studies subcommittees of the Durham Veterans Affairs Medical Center and the Central Texas Veterans Health Care System.

### Induction of AS Activity in Middle-aged Rats

Acute seizure activity in middle-aged rats was induced through graded intraperitoneal (i.p.) injections of KA, as described in our previous studies [46], [47]. Intraperitoneal injections of KA were administered to animals every hour at a dose of 3.0-mg/Kg b.w. until they developed continuous AS activity or status epilepticus (SE). Induction of continuous AS activity in middle-aged rats required 2–4 hourly injections of KA. Thus, each animal received a total KA dose of 6–12.0 mg/Kg b.w. Seizures were scored as described in our earlier report [46]. The stages III–V motor seizures were typified by unilateral forelimb clonus with lordotic posture (stage III), bilateral forelimb clonus and rearing (stage IV) and bilateral forelimb clonus with rearing and falling (stage V). The onset of continuous AS activity was defined as the first stage IV or V seizure that did not decline after several minutes. All animals receiving the above doses of KA exhibited continuous stages III–V motor seizures for >4 hours after the onset of AS activity. We quantified the number of motor seizures (stages III–V) and the duration of individual seizures for each hour during the period of SE. Rats that underwent AS activity were given moistened rat chow and a 5–10 ml injection of lactated Ringer’s solution every day for 3–5 days. Out of 8 rats that underwent AS activity, two rats died during the survival period after the induction of AS activity and one rat was excluded as it exhibited seizures for less than three hours. The remaining 5 rats were used for further studies as described below.

### Labeling of Newly Born Cells in the DG and Tissue Processing

Rats belonging to both control group and the group that underwent AS activity received daily i.p. injections of BrdU (Sigma, St Louis, MO) for 12 consecutive days, at a dose of 100-mg/kg b.w. In rats that underwent AS activity, daily BrdU injections commenced on the day of AS activity and ended on post-seizure day 12. Rats were fatally anesthetized with isoflurane and perfused with 4% paraformaldehyde at 24 hours after the last of twelve daily BrdU injections and brains were collected. The brains were next post-fixed in 4% paraformaldehyde for 16 hours at 4°C and cryoprotected in 30% sucrose solution in phosphate buffer (PB). Thirty-micrometer thick cryostat sections were cut coronally through the entire antero-posterior axis of the hippocampus and collected serially in PB. Every 15^th^ section through the hippocampus was selected in each of the animals and processed for Nissl staining to visualize the hippocampal cytoarchitecture as well as the extent of hippocampal injury after AS activity. Nissl staining was accomplished through histological processing of serial sections mounted on glass slides for hydration, cresyl violet treatment, dehydration and clearing.

### Measurement of Numbers of BrdU+ Cells and DCX+ Neurons in the DG

A set of serial sections (every 15^th^) through the entire septo-temporal axis of the hippocampus were selected in each animal belonging to the two groups and processed for BrdU immunostaining using a monoclonal antibody to BrdU (Roche; Indianapolis, IN). Another series (every 15^th^) of sections from animals were processed for DCX immunostaining using a polyclonal antibody to DCX (Santa Cruz Biotechnology; Santa Cruz, CA) using the avidin-biotin complex method, as described in our earlier reports [27], [28], [50]. The BrdU+ cells in the dentate SGZ (two-cell thick region from the inner margin of the dentate GCL) and the GCL were counted in every 15^th^ section through the entire antero-posterior extent of the hippocampus in every animal belonging to the two groups (n = 5/group). Furthermore, DCX+ neurons in the SGZ-GCL and the DH were counted separately in animals belonging to the two groups. The StereoInvestigator system (Microbrightfield Inc., Williston, VT), consisting of a color digital video camera (Optronics Inc., Muskogee, OK) interfaced with a Nikon E600 microscope was used for counting of cells [27], [28], [51]. In brief, cells were counted from 50–400 randomly and systematically selected frames (each measuring 40×40 µm) in every selected section using a 100X oil immersion objective lens. The numbers and densities of counting frames were determined by entering grid size in the optical fractionator component of the StereoInvestigator system. For cell counts in every chosen section, the contour of SGZ-GCL area was first delineated using the tracing function. The optical fractionator component was then activated, and numbers and locations of counting frames and the depth for counting was determined by entering parameters such as grid size, thickness of the top guard zone (4 µm) and the optical dissector height (i.e. 8 µm). A computer driven motorized stage then allowed the section to be analyzed at each of the counting frame locations. All cells that came into focus in the middle 8-µm section thickness were counted if they were entirely within the counting frame or touching the upper or right side of the counting frame. The StereoInvestigator program then calculated the total number of BrdU+/DCX+ cells per each chosen region (SGZ-GCL or the DH) by utilizing the optical fractionator formula, as described in our earlier reports [27], [28], [51].

### Analyses of Neuronal Fate-choice Decision of Newly Born Cells

For measuring the percentages of newly born cells (BrdU+ cells) that differentiated into DCX+ neurons in the SGZ-GCL, we processed serial sections from animals belonging to both groups for DCX and BrdU dual immunofluorescence, as described in our earlier reports [28], [51]. Cells that exhibited BrdU and DCX co-expression were identified using a confocal microscope. Fractions of BrdU+ cells that expressed DCX were quantified by examination of individual BrdU+ cells via one-micrometer thick optical Z-sections sampled from different regions of the SGZ-GCL.

### Statistical Analyses

For every parameter, the average value was first calculated separately for each animal before the means and standard errors were determined for the total number of animals included per group. The values (Mean ± S.E.M.) from two groups of animals were compared using a two-tailed, unpaired Student’s t-test.

## References

[1] Altman J, Das GD (1965) Autoradiographic and histological evidence of postnatal hippocampal neurogenesis in rats. J Comp Neurol 124: 319–335. 5861717 10.1002/cne.901240303

[2] Gould E, McEwen BS, Tanapat P, Galea LA, Fuchs E (1997) Neurogenesis in the dentate gyrus of the adult tree shrew is regulated by psychosocial stress and NMDA receptor activation. J Neurosci 17: 2492–2498. 9065509 10.1523/JNEUROSCI.17-07-02492.1997 PMC6573503

[3] Eriksson PS, Perfilieva E, Bjork-Eriksson T, Alborn AM, Nordborg C, et al (1998) Neurogenesis in the adult human hippocampus. Nat Med 4: 1313–1317. 9809557 10.1038/3305

[4] Gould E, Reeves AJ, Fallah M, Tanapat P, Gross CG, et al (1999) Hippocampal neurogenesis in adult Old World primates. Proc Natl Acad Sci USA 96: 5263–5267. 10220454 10.1073/pnas.96.9.5263 PMC21852

[5] Santarelli L, Saxe M, Gross C, Surget A, Battaglia F, et al (2003) Requirement of hippocampal neurogenesis for the behavioral effects of antidepressants. Science 301: 805–809. 12907793 10.1126/science.1083328

[6] Leuner B, Gould E, Shors TJ (2006) Is there a link between adult neurogenesis and learning? Hippocampus 16: 216–224. 16421862 10.1002/hipo.20153

[7] Kee N, Teixeira CM, Wang AH, Frankland PW (2007) Preferential incorporation of adult-generated granule cells into spatial memory networks in the dentate gyrus. Nat Neurosci 10: 355–362. 17277773 10.1038/nn1847

[8] Dupret D, Revest JM, Koehl M, Ichas F, De Giorgi F, et al (2008) Spatial relational memory requires hippocampal adult neurogenesis. PLoS One 3: e1959. 18509506 10.1371/journal.pone.0001959 PMC2396793

[9] Clelland CD, Choi M, Romberg C, Clemenson GD, Fragniere A, et al (2009) A functional role for adult hippocampal neurogenesis in spatial pattern separation. Science 325: 210–213. 19590004 10.1126/science.1173215 PMC2997634

[10] Imayoshi I, Sakamoto M, Ohtsuka T, Takao K, Miyakawa T, et al (2008) Roles of continuous neurogenesis in the structural and functional integrity of the adult forebrain. Nat Neurosci 11: 1153–1161. 18758458 10.1038/nn.2185

[11] Jessberger S, Clark RE, Broadbent NJ, Clemenson GD Jr, Consiglio A, et al (2009) Dentate gyrus-specific knockdown of adult neurogenesis impairs spatial and object recognition memory in adult rats. Learn Mem 16: 147–154. 19181621 10.1101/lm.1172609 PMC2661246

[12] Deng W, Aimone JB, Gage FH (2010) New neurons and new memories: how does adult hippocampal neurogenesis affect learning and memory? Nat Rev Neurosci 11: 339–350. 20354534 10.1038/nrn2822 PMC2886712

[13] Kempermann G, Brandon EP, Gage FH (1998) Environmental stimulation of 129/SvJ mice causes increased cell proliferation and neurogenesis in the adult dentate gyrus. Curr Biol 8: 939–942. 9707406 10.1016/s0960-9822(07)00377-6

[14] Kempermann G, Gast D, Gage FH (2002) Neuroplasticity in old age: sustained fivefold induction of hippocampal neurogenesis by long-term environmental enrichment. Ann Neurol 52: 135–143. 12210782 10.1002/ana.10262

[15] Gould E (1999) Serotonin and hippocampal neurogenesis. Neuropsychopharmacology 21: 46S–51S. 10432488 10.1016/S0893-133X(99)00045-7

[16] Nilsson M, Perfilieva E, Johansson U, Orwar O, Eriksson PS (1999) Enriched environment increases neurogenesis in the adult rat dentate gyrus and improves spatial memory. J Neurobiol 39: 569–578. 10380078 10.1002/(sici)1097-4695(19990615)39:4<569::aid-neu10>3.0.co;2-f

[17] van Praag H, Kempermann G, Gage FH (1999) Running increases cell proliferation and neurogenesis in the adult mouse dentate gyrus. Nat Neurosci 2: 266–270. 10195220 10.1038/6368

[18] van Praag H, Shubert T, Zhao C, Gage FH (2005) Exercise enhances learning and hippocampal neurogenesis in aged mice. J Neurosci 25: 8680–8685. 16177036 10.1523/JNEUROSCI.1731-05.2005 PMC1360197

[19] Lichtenwalner RJ, Forbes ME, Bennett SA, Lynch CD, Sonntag WE, et al (2001) Intracerebroventricular infusion of insulin-like growth factor-I ameliorates the age-related decline in hippocampal neurogenesis. Neuroscience 107: 603–613. 11720784 10.1016/s0306-4522(01)00378-5

[20] Jin K, Sun Y, Xie L, Batteur S, Mao XO, et al. (2003) Neurogenesis and aging: FGF-2 and HB-EGF restore neurogenesis in hippocampus and subventricular zone of aged mice. Aging Cell 2, 175–183. 10.1046/j.1474-9728.2003.00046.x 12882410

[21] Jin K, Zhu Y, Sun Y, Mao XO, Xie L, et al (2002) Vascular endothelial growth factor (VEGF) stimulates neurogenesis in vitro and in vivo. Proc Natl Acad Sci USA 99: 11946–11950. 12181492 10.1073/pnas.182296499 PMC129374

[22] Sun Y, Jin K, Childs JT, Xie L, Mao XO, et al (2006) Vascular endothelial growth factor-B (VEGFB) stimulates neurogenesis: evidence from knockout mice and growth factor administration. Dev Biol 289: 329–335. 16337622 10.1016/j.ydbio.2005.10.016

[23] Sun Y, Jin K, Xie L, Childs J, Mao XO, et al (2003) VEGF-induced neuroprotection, neurogenesis, and angiogenesis after focal cerebral ischemia. J Clin Invest 111: 1843–1851. 12813020 10.1172/JCI17977 PMC161428

[24] Mirescu C, Peters JD, Gould E (2004) Early life experience alters response of adult neurogenesis to stress. Nat Neurosci 7: 841–846. 15273691 10.1038/nn1290

[25] Rai K, Hattiangady B, Shetty AK (2007) Enhanced production and dendritic growth of new dentate granule cells in the middle-aged and aged hippocampus following intracerebroventricular FGF-2 infusions. Eur J Neurosci 26: 1765–1779. 17883411 10.1111/j.1460-9568.2007.05820.x

[26] Kuhn HG, Dickinson-Anson H, Gage FH (1996) Neurogenesis in the dentate gyrus of the adult rat: age-related decrease of neuronal progenitor proliferation. J Neurosci 16: 2027–2033. 8604047 10.1523/JNEUROSCI.16-06-02027.1996 PMC6578509

[27] Rao MS, Hattiangady B, Abdel-Rahman A, Stanley DP, Shetty AK (2005) Newly born cells in the ageing dentate gyrus display normal migration, survival and neuronal fate choice but endure retarded early maturation. Eur J Neurosci 21: 464–476. 15673445 10.1111/j.1460-9568.2005.03853.x

[28] Rao MS, Hattiangady B, Shetty AK (2006) The window and mechanisms of major age-related decline in the production of new neurons within the dentate gyrus of the hippocampus. Aging Cell 5: 545–558. 17129216 10.1111/j.1474-9726.2006.00243.x

[29] Shetty AK, Hattiangady B, Shetty GA (2005) Stem/progenitor cell proliferation factors FGF-2, IGF-1, and VEGF exhibit early decline during the course of aging in the hippocampus: role of astrocytes. Glia 51: 173–86. 15800930 10.1002/glia.20187

[30] Hattiangady B, Shetty AK (2008) Aging does not alter the number or phenotype of putative stem/progenitor cells in the neurogenic region of the hippocampus. Neurobiol Aging 29: 129–147. 17092610 10.1016/j.neurobiolaging.2006.09.015 PMC3612500

[31] Encinas JM, Michurina TV, Peunova N, Park JH, Tordo J, et al (2011) Division-coupled astrocytic differentiation and age-related depletion of neural stem cells in the adult hippocampus. Cell Stem Cell 8: 566–579. 21549330 10.1016/j.stem.2011.03.010 PMC3286186

[32] Liu J, Solway K, Messing RO, Sharp FR (1998) Increased neurogenesis in the dentate gyrus after transient global ischemia in gerbils. J Neurosci 18: 7768–7778. 9742147 10.1523/JNEUROSCI.18-19-07768.1998 PMC6793017

[33] Jin K, Minami M, Lan JQ, Mao XO, Batteur S, et al (2001) Neurogenesis in dentate subgranular zone and rostral subventricular zone after focal cerebral ischemia in the rat. Proc Natl Acad Sci USA 98: 4710–4715. 11296300 10.1073/pnas.081011098 PMC31899

[34] Jin K, Minami M, Xie L, Sun Y, Mao XO, et al (2004) Ischemia-induced neurogenesis is preserved but reduced in the aged rodent brain. Aging Cell 3: 373–377. 15569354 10.1111/j.1474-9728.2004.00131.x

[35] Parent JM, Yu TW, Leibowitz RT, Geschwind DH, Sloviter RS, et al (1997) Dentate granule cell neurogenesis is increased by seizures and contributes to aberrant network reorganization in the adult rat hippocampus. J Neurosci 17: 3727–3738. 9133393 10.1523/JNEUROSCI.17-10-03727.1997 PMC6573703

[36] Gray WP, Sundstrom LE (1998) Kainic acid increases the proliferation of granule cell progenitors in the dentate gyrus of the adult rat. Brain Res 790: 52–59. 9593820 10.1016/s0006-8993(98)00030-4

[37] Hattiangady B, Rao MS, Shetty AK (2004) Chronic temporal lobe epilepsy is associated with severely declined dentate neurogenesis in the adult hippocampus. Neurobiol Dis 17: 473–490. 15571983 10.1016/j.nbd.2004.08.008

[38] Hattiangady B, Rao MS, Shetty AK (2008) Plasticity of hippocampal stem/progenitor cells to enhance neurogenesis in response to kainate-induced injury is lost by middle age. Aging Cell 7: 207–224. 18241325 10.1111/j.1474-9726.2007.00363.x PMC3612497

[39] Overstreet-Wadiche LS, Bromberg DA, Bensen AL, Westbrook GL (2006) Seizures accelerate functional integration of adult-generated granule cells. J Neurosci 26: 4095–4103. 16611826 10.1523/JNEUROSCI.5508-05.2006 PMC6673901

[40] Kleindienst A, McGinn MJ, Harvey HB, Colello RJ, Hamm RJ, et al (2005) Enhanced hippocampal neurogenesis by intraventricular S100B infusion is associated with improved cognitive recovery after traumatic brain injury. J Neurotrauma 22: 645–655. 15941374 10.1089/neu.2005.22.645

[41] Sun D, McGinn MJ, Zhou Z, Harvey HB, Bullock MR, et al (2007) Anatomical integration of newly generated dentate granule neurons following traumatic brain injury in adult rats and its association to cognitive recovery. Exp Neurol 204: 264–272. 17198703 10.1016/j.expneurol.2006.11.005

[42] Jessberger S, Nakashima K, Clemenson GD Jr, Mejia E, Mathews E, et al (2007) Epigenetic modulation of seizure-induced neurogenesis and cognitive decline. J Neurosci 27: 5967–5975. 17537967 10.1523/JNEUROSCI.0110-07.2007 PMC6672253

[43] Scharfman HE, Gray WP (2007) Relevance of seizure-induced neurogenesis in animal models of epilepsy to the etiology of temporal lobe epilepsy. Epilepsia 48 Suppl 233–41. 10.1111/j.1528-1167.2007.01065.x PMC2504501 17571351

[44] Jakubs K, Nanobashvili A, Bonde S, Ekdahl CT, Kokaia Z, et al (2006) Environment matters: synaptic properties of neurons born in the epileptic adult brain develop to reduce excitability. Neuron 52: 1047–1059. 17178407 10.1016/j.neuron.2006.11.004

[45] Pekcec A, Mühlenhoff M, Gerardy-Schahn R, Potschka H (2007) Impact of the PSA-NCAM system on pathophysiology in a chronic rodent model of temporal lobe epilepsy. Neurobiol Dis. 27: 54–66. 10.1016/j.nbd.2007.04.002 17513116

[46] Rao MS, Hattiangady B, Shetty AK (2008) Status epilepticus during old age is not associated with enhanced hippocampal neurogenesis. Hippocampus 18: 931–944. 18493929 10.1002/hipo.20449 PMC3612499

[47] Hattiangady B, Kuruba R, Shetty AK (2011) Acute seizures in old age leads to a greater loss of CA1 pyramidal neurons, an increased propensity for developing chronic TLE and a severe cognitive dysfunction. Aging Dis 2: 1–17. 21339903 PMC3041587

[48] Woods AG, Guthrie KM, Kurlawalla MA, Gall CM (1998) Deafferentation-induced increases in hippocampal insulin-like growth factor-1 messenger RNA expression are severely attenuated in middle aged and aged rats. Neuroscience 83: 663–668. 9483550 10.1016/s0306-4522(97)00539-3

[49] Shetty AK, Rao MS, Hattiangady B, Zaman V, Shetty GA (2004) Hippocampal neurotrophin levels after injury: Relationship to the age of the hippocampus at the time of injury. J Neurosci Res 78: 520–532. 15468179 10.1002/jnr.20302

[50] Rao MS, Shetty AK (2004) Efficacy of doublecortin as a marker to analyse the absolute number and dendritic growth of newly generated neurons in the adult dentate gyrus. Eur J Neurosci 19: 234–246. 14725617 10.1111/j.0953-816x.2003.03123.x

[51] Shetty AK, Hattiangady B, Rao MS, Shuai B (2011) Deafferentation enhances neurogenesis in the young and middle aged hippocampus but not in the aged hippocampus. Hippocampus 21: 631–646. 20333732 10.1002/hipo.20776 PMC2927723

[52] Rao MS, Hattiangady B, Reddy DS, Shetty AK (2006) Hippocampal neurodegeneration, spontaneous seizures, and mossy fiber sprouting in the F344 rat model of temporal lobe epilepsy. J Neurosci Res 83: 1088–1105. 16493685 10.1002/jnr.20802

[53] Kempermann G, Gast D, Kronenberg G, Yamaguchi M, Gage FH (2003) Early determination and long-term persistence of adult-generated new neurons in the hippocampus of mice. Development 130: 391–399. 12466205 10.1242/dev.00203

[54] Shapiro LA, Ribak CE (2006) Newly born dentate granule neurons after pilocarpine-induced epilepsy have hilar basal dendrites with immature synapses. Epilepsy Res 69: 53–66. 16480853 10.1016/j.eplepsyres.2005.12.003

[55] Jessberger S, Zhao C, Toni N, Clemenson GD Jr, Li Y, et al (2007b) Seizure-associated, aberrant neurogenesis in adult rats characterized with retrovirus-mediated cell labeling. J Neurosci 27: 9400–9407. 17728453 10.1523/JNEUROSCI.2002-07.2007 PMC6673128

[56] Shetty AK, Zaman V, Hattiangady B (2005) Repair of the injured adult hippocampus through graft-mediated modulation of the plasticity of the dentate gyrus in a rat model of temporal lobe epilepsy. J Neurosci 25: 8391–8401. 16162921 10.1523/JNEUROSCI.1538-05.2005 PMC6725675

[57] Dudek FE, Sutula TP (2007) Epileptogenesis in the dentate gyrus: a critical perspective. Prog Brain Res 163: 755–73. 17765749 10.1016/S0079-6123(07)63041-6

[58] Shetty AK, Turner DA (2000) Fetal hippocampal grafts containing CA3 cells restore host hippocampal glutamate decarboxylase-positive interneuron numbers in a rat model of temporal lobe epilepsy. J Neurosci 20: 8788–8801. 11102487 10.1523/JNEUROSCI.20-23-08788.2000 PMC6773070

[59] Ben-Ari Y (2006) Seizures beget seizures; the quest for GABA as a key player. Crit Rev Neurobiol 18, 135–144. 10.1615/critrevneurobiol.v18.i1-2.140 17725516

[60] Kuruba R, Hattiangady B, Parihar VK, Shuai B, Shetty AK (2011) Differential susceptibility of interneurons expressing neuropeptide Y or parvalbumin in the aged hippocampus to acute seizure activity. PLoS One 6: e24493. 21915341 10.1371/journal.pone.0024493 PMC3167860

[61] De Lanenrolle NC, Lee T-S, Spencer DD (2010) Astrocytes and epilepsy. Neurotherapeutics 7: 424–438. 20880506 10.1016/j.nurt.2010.08.002 PMC5084304

[62] Vezzani A, Aronica E, Mazarati A, Pittman QJ (2011) Epilepsy and brain inflammation. Exp Neurol. doi: 10.1016/j.expneurol.2011.09.033. 10.1016/j.expneurol.2011.09.033 21985866

[63] Murphy BL, Pun RY, Yin H, Faulkner CR, Loepke AW, et al (2011) Heterogeneous integration of adult-generated granule cells into the epileptic brain. J Neurosci 31: 105–117. 21209195 10.1523/JNEUROSCI.2728-10.2011 PMC3022369

[64] Shetty AK, Hattiangady B (2007) Prospects of Stem Cell Therapy for Temporal Lobe Epilepsy. Stem Cells 25: 2396–407. 17600108 10.1634/stemcells.2007-0313 PMC3612506

[65] Dashtipour K, Tran PH, Okazaki MM, Nadler JV, Ribak CE (2001) Ultrastructural features and synaptic connections of hilar ectopic granule cells in the rat dentate gyrus are different from those of granule cells in the granule cell layer. Brain Res 890: 261–271. 11164792 10.1016/s0006-8993(00)03119-x

[66] Pierce JP, Melton J, Punsoni M, McCloskey DP, Scharfman HE (2005) Mossy fibers are the primary source of afferent input to ectopic granule cells that are born after pilocarpine-induced seizures. Exp Neurol 196: 316–331. 16342370 10.1016/j.expneurol.2005.08.007 PMC1431686

[67] Cameron MC, Zhan RZ, Nadler JV (2011) Morphologic integration of hilar ectopic granule cells into dentate gyrus circuitry in the pilocarpine model of temporal lone epilepsy. J Comp Neurol 519: 2175–2192. 21455997 10.1002/cne.22623 PMC3908827

[68] Scharfman HE, Goodman JH, Sollas AL (2000) Granule-like neurons at the hilar/CA3 border after status epilepticus and their synchrony with area CA3 pyramidal cells: functional implications of seizure-induced neurogenesis. J Neurosci 20: 6144–6158. 10934264 10.1523/JNEUROSCI.20-16-06144.2000 PMC6772593

[69] Scharfman HE, Smith KL, Goodman JH, Sollas AL (2001) Survival of dentate hilar mossy cells after pilocarpine-induced seizures and their synchronized burst discharges with area CA3 pyramidal cells. Neuroscience 104: 741–759. 11440806 10.1016/s0306-4522(01)00132-4 PMC2518406

[70] McCloskey DP, Hintz TM, Pierce JP, Scharfman HE (2006) Stereological methods reveal the robust size and stability of ectopic hilar granule cells after pilocarpine-induced status epilepticus in the adult rat. Eur J Neurosci 24: 2203–10. 17042797 10.1111/j.1460-9568.2006.05101.x PMC3924324

[71] Jung KH, Chu K, Kim M, Jeong SW, Song YM, et al (2004) Continuous cytosine-b-D-arabinofuranoside infusion reduces ectopic granule cells in adult rat hippocampus with attenuation of spontaneous recurrent seizures following pilocarpine-induced status epilepticus. Eur J Neurosci 19: 3219–3226. 15217378 10.1111/j.0953-816X.2004.03412.x

[72] Mattiesen WR, Tauber SC, Gerber J, Bunkowski S, Bruck W, et al (2009) Increased neurogenesis after hypoxic-ischemic encephalopathy in humans is age-related. Acta Neuropathol 117: 525–535. 19277687 10.1007/s00401-009-0509-0

[73] Walter J, Keiner S, Witte OW, Redecker C (2010) Differential stroke-induced proliferative response of distinct precursor cell subpopulations in the young and aged dentate gyrus. Neuroscience 169: 1279–1286. 20570606 10.1016/j.neuroscience.2010.05.035

[74] Shapiro LA, Wang L, Upadhyaya P, Ribak CF (2011) Seizure-induced increased neurogenesis occurs in the dentate gyrus of aged Sprague-Dawley rats. Aging Dis 2: 286–293. 22396880 PMC3295072

[75] Tan YF, Preston E, Wojtowicz JM (2010) Enhanced post-ischemic neurogenesis in aging rats. Front Neurosci 4: pii: 163. 10.3389/fnins.2010.00163 PMC2944628 20877422

[76] Sivilia S, Giuliani A, Del Vecchio G, Giardino L, Calza L (2008) Age-dependent impairment of hippocampal neurogenesis in chronic cerebral hypoperfusion. Neuropathol Appl Neurobiol 34: 52–61. 17931356 10.1111/j.1365-2990.2007.00863.x

